# Access to water, sanitation and hygiene services in health facilities in sub-Saharan Africa 2013–2018: Results of health facility surveys and implications for COVID-19 transmission

**DOI:** 10.1186/s12913-021-06515-z

**Published:** 2021-06-25

**Authors:** Mufaro Kanyangarara, Savannah Allen, Safia S Jiwani, David Fuente

**Affiliations:** 1 Department of Epidemiology and Biostatistics, Arnold School of Public Health, University of South Carolina, 915 Greene Street, SC 29201 Columbia, USA; 2 Department of International Health, Johns Hopkins Bloomberg School of Public Health, Baltimore, MD USA; 3 School of Earth, Ocean and the Environment, University of South Carolina, Columbia, SC USA

**Keywords:** Water, Sanitation and hygiene, Health care facilities, Coronavirus disease (COVID-19), Inequalities, Sub-Saharan Africa

## Abstract

**Background:**

The COVID-19 pandemic has highlighted important needs in water, sanitation and hygiene (WASH) services and standard practices for infection prevention and control in sub-Saharan Africa. We assessed the availability of WASH and standard precautions for infection prevention in health facilities across 18 countries in sub-Saharan Africa, as well as inequalities by location (rural/urban) and managing authority (public/private). Data from health facility surveys conducted between 2013 and 2018 in 18 sub-Saharan African countries were used to estimate the access to an improved water source within 500 m, an improved toilet, soap and running water or alcohol-based hand rub, and standard precautions for infection prevention at health facilities. Rural-urban differences and public-private differences in access to services were calculated. We also compared population level access to health facility access to services.

**Result:**

Overall, 16,456 health facilities from 18 countries were included. Across countries, an estimated 88 % had an improved water source, 94 % had an improved toilet, 74 % had soap and running water or alcohol-based hand rub, and 17 % had standard precautions for infection prevention available. There was wide variability in access to water, sanitation and hygiene services between rural and urban health facilities and between public and private facilities, with consistently lower access in both rural and public facilities. In both rural and urban areas, access to water, sanitation and hygiene services was ubiquitously better at health facilities than households.

**Conclusions:**

Availability of WASH services in health facilities in sub-Saharan Africa has improved but remains below the global target of 80 % in many countries. Ensuring adequate access to WASH services and enforcing adherence to safety and hygiene practices in health facilities will be essential to minimize the risk of COVID-19 transmission.

## Introduction

Since 2000, significant strides have been made to improve access to clean and safe water, sanitation, and hygiene (WASH) in households. Sub-Saharan Africa recorded notable gains, with 328 million people gaining access to basic drinking water and 163 million people gaining access to basic sanitation services since 2000 [1]. Nevertheless, 400 million people in the region still lack access to basic drinking water and 709 million lack access to basic sanitation services [1]. In most countries in the region, less than half of the population has a handwashing facility with soap and water available at home [2]. Furthermore, pronounced inequalities in access to WASH by gender, socioeconomic status and rural-urban residence persist [1, 3]. Consistent with disparities observed in access to other essential services, households in rural areas and urban slums have relatively poorer access to improved water and sanitation facilities compared to urban households [4–7]. To address these gaps, the Sustainable Development Goal (SDG) 6 aims to achieve universal and equitable access to safe affordable drinking water, sanitation, and hygiene for all by 2030 [8]. Improving access to WASH reduces neonatal and child mortality and the burden of infectious diseases, and improves nutritional status, girl’s education, and the overall quality of life [9–11]. Expanding access to WASH services is inextricably linked to progress on SDGs related to health, nutrition, education, and gender equality. A recent assessment of the feasibility of attaining near universal coverage of basic WASH services indicated that less than one third of 132 countries are on track to reach this goal by 2030 [1]. Substantial variation in progress exists both within and between countries, and accelerated progress is needed to meet the WASH-related targets in most countries in sub-Saharan Africa.

While access to essential health services in sub-Saharan Africa has improved in recent years [12], the quality of care received during contact with the health system remains insufficient to improve health outcomes [13, 14]. Health facilities lack the necessary infrastructure, equipment, medicines, commodities, and trained personnel to create an enabling environment, resulting in missed opportunities to provide quality essential health services [15, 16]. While about one fifth of deaths occurring in low- and middle-income countries (LMICs) are attributable to the lack of access to health services, one third of deaths are a result of the receipt of poor quality of care which is often linked to insufficient readiness of facilities to provide services [17].

Structural inputs such as WASH services are a prerequisite for the delivery of quality essential health services. For instance, clean birth practices including handwashing by birth attendants, cleaning the maternal perineum, use of a clean birth surface, clean cutting and tying of the cord, and hygienic cord and skin care immediately after delivery, can reduce neonatal sepsis deaths by 27 %, neonatal tetanus deaths by 38 % and maternal sepsis deaths by 60 % [18, 19]. The lack of WASH services in health facilities increases the risk of healthcare associated infections and lowers patient satisfaction with services leading to delays in care-seeking, hindering the provision of quality essential health services and the attainment of sustainable development goals [20]. Despite the importance of WASH, an estimated 51 % of health facilities across sub-Saharan Africa have basic water service and 23 % have basic sanitation services [21]. Achieving universal access to WASH in health facilities is key to achieving universal access to quality care.

Effective WASH is also necessary to prevent and control the spread of coronavirus disease 2019 (COVID-19). The current COVID-19 pandemic has highlighted deficiencies in access to WASH services in health facilities and underscored the need for increased political commitment and enhanced accountability to address WASH gaps in health facilities. As of April 28, 2021, sub-Saharan Africa had over 4.5 million cases and 120,000 deaths from COVID-19 [22]. The continent accounts for less than 4 % of the global burden of COVID-19 cases and deaths [22]. The relatively lower burden of COVID-19 cases and deaths compared to other regions has been attributed to the younger population, prior exposure to other coronaviruses, limited testing capacity, weak surveillance systems, and the rapid implementation and enforcement of lockdowns and other mitigation measures [23]. Nevertheless the COVID-19 pandemic raises concerns about the ability of sub-Saharan African countries to mount an effective pandemic response and recovery plan, given the role of WASH in the prevention of COVID-19 infection and transmission at the community-level. Interim guidance from the World Health Organization (WHO) on WASH and waste management practices in health facilities recommends frequent hand hygiene, regular environmental cleaning and disinfection, safe management of excreta and healthcare waste during the pandemic [24]. Emerging reports suggest shortages of personal protective equipment (PPE), and inadequate WASH services, waste management services and standard precautions for infection control, which are critical in reducing disease transmission [23, 25]. While several studies have discussed the crucial role of WASH during the pandemic, few studies have provided systematic data on the current situation in health facilities is sparse [26]. Data on the availability of WASH services prior to the COVID-19 pandemic can inform immediate policy actions and support ongoing efforts to curb the transmission of COVID-19 through improvements in access. The overall goal of this study is to assess the availability of WASH services and infection prevention and control measures in health facilities across sub-Saharan Africa. The findings presented complement previous regional and national estimates of the availability of WASH in health facilities by focusing on indicators most relevant to COVID-19 and unmasking inequalities by rural-urban location and managing authority (public/private). Assessment of both population and health facility access to WASH services further sheds light on the relatively worse situation in rural areas.

## Methods

Country-specific data on the availability of WASH services and standard precautions for infection control in health facilities across sub-Saharan Africa were obtained from the Service Availability and Readiness Assessment (SARA) and the Service Provision Assessment (SPA) surveys. The SPA and SARA surveys are comprehensive health system assessments that are implemented in over 40 LMICs [27, 28]. Typically, stratified random sampling is used to select a nationally representative sample of health facilities from a master health facility list containing all public and private (including local and international non-governmental and faith-based) health facilities. The availability and functionality of essential components spanning 5 domains is observed and documented: amenities, equipment, standard precautions for infection control, diagnostic capacity, and medicines. Further details on the sampling and data collection procedures used in the SPA and SARA surveys are detailed elsewhere [27, 28].

**Fig. 1:**
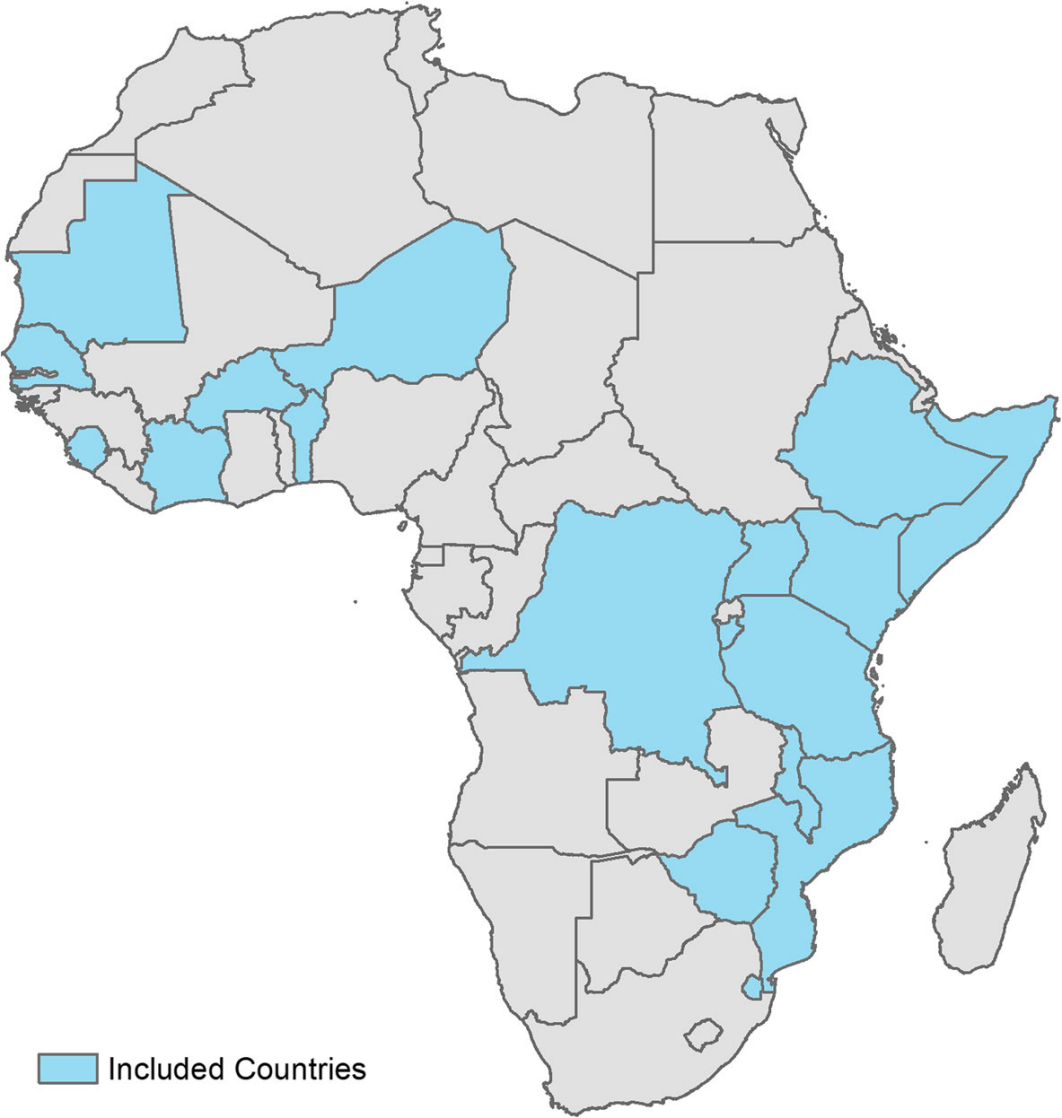
Map of African countries included in analysis

We included data from Sub-Saharan African countries with an available SPA or SARA survey conducted in the last 7 years. Given the limited availability of recent data, this study period was selected to allow for geographic representation from the inclusion of more countries. For countries with multiple surveys available within the study period, we used the most recent survey. Based on this criteria, 18 countries were included in the analysis: Benin (2015), Burkina Faso (2018), Burundi (2017), Cote d’Ivoire (2016), Democratic Republic of Congo (2017-18), Eswatini (2017), Ethiopia (2018), Kenya (2018), Malawi (2013-14), Mauritania (2018), Mozambique (2018), Niger (2015), Senegal (2018), Sierra Leone (2017), Somalia (2016), Tanzania 2014-15), Uganda (2013), and Zimbabwe (2015) (Fig. 1). The 2020 population for the countries included in the study is projected to be 578 million, accounting for 52 % of the population of sub-Saharan Africa [29].The rural population in the 18 countries ranges from 13 % in Burundi to 55 % in the Democratic Republic of Congo (Table 1) [30]. As a proxy for access to essential health services, the percentage of deliveries occurring in health facilities was considered; the median across countries was 77 %, indicating fairly high levels of access to care [31].

**Table 1:** Characteristics of included countries

Code	Country	Year	Survey	Number of health facilities	Total Country Population (Thousands)^a^	Urban Population (%)^b^	Government expenditure on health as % of GDP^3^	Health facility delivery rate (%)^c^
BEN	Benin	2015	SARA	788	12,123	48	0.8	84
BFA	Burkina Faso	2018	SARA	794	20,903	30	1.7	82
BDI	Burundi	2017	SARA	206	11,891	13	2.5	84
CIV	Cote d’Ivoire	2016	SARA	963	26,378	51	1.1	70
COD	DRC	2017-18	SPA	1,555	89,561	45	0.7	80
SWZ	Eswatini	2017	SARA	327	1,160	24	**-**	88
ETH	Ethiopia	2018	SARA	764	114,964	21	5.0	26
KEN	Kenya	2018	SARA	2,927	53,771	28	1.7	61
MWI	Malawi	2013-14	SPA	1,060	19,130	17	2.7	91
MRT	Mauritania	2018	SARA	919	4,650	55	1.8	69
MOZ	Mozambique	2018	SARA	1,643	31,255	37	0.4	55
NER	Niger	2015	SARA	372	24,207	17	1.5	59
SEN	Senegal	2018	SPA	339	16,744	48	1.7	78
SLE	Sierra Leone	2017	SARA	1,284	7,977	42	1.6	77
SOM	Somalia	2016	SARA	799	15,893	46	**-**	9
TZA	Tanzania	2014-15	SPA	1,188	59,734	35	**-**	**-**
UGA	Uganda	2013	SARA	209	45,741	24	1.0	73
ZWE	Zimbabwe	2015	SARA	275	14,863	32	4.0	77

**–** indicates data not available. *DRC* Democratic Republic of Congo, *SARA* Service Availability and Readiness Assessment, *SPA* Service Provision Assessment

^a^ Source: United Nations. World Population Prospects 2019 [29]

^b^ Source: United Nations Population Division. Urban population. 2018 [30]

^c^ Source: UNICEF. The State of the World’s Children 2019: Children, Food and Nutrition: Growing Well in a Changing World. UNICEF; 2019 [31]

To assess the availability of WASH and standard precautions for infection prevention, indicators relevant to the COVID-19 pandemic response were selected from the World Health Organization (WHO) indicators used to assess general service readiness (Table 2) [28]. Indicators were defined based on the presence of tracer items on the day of the health facility assessment. Availability of water service was defined as the presence of an improved water supply within 500m and sanitation service was defined as the presence of an improved toilet on the premises. Definitions of “improved” were based on the improved/unimproved facility type classification introduced during the Millennium Development Goals era [1]. Hygiene services was based on access to soap and running water or alcohol-based rub. Availability of standard precautions for infection prevention was assessed by considering the presence of the following tracer items: appropriate storage and safe final disposal of sharps and other infectious wastes, guidelines for standard precautions, environmental disinfectant, latex gloves, single use-standard disposable or auto-disable syringes, and soap and water or alcohol-based hand rub. A health facility was considered to have standard precautions for infection prevention if all tracer items were available on the day of assessment.

**Table 2:** Definition of indicators

Domain	Indicator	Definition
Water services	Health facilities with an improved water supply within 500 m	Piped, public tap, standpipe, tubewell/borehole, protected dug well, protected spring, rain water.
Sanitation services	Health facilities with improved toilets	Observed availability of flush/pour flush to piped sewer system or septic tank or pit latrine, pit latrine (ventilated improved pit (VIP) or other) with slab, composting toilet.
Hand hygiene services	Health facilities with soap and running water/ alcohol-based hand rub available	Observed availability of soap and running water or alcohol-based hand rub
Standard precautions for infection prevention	Health facilities with:	
	safe final disposal of infectious wastes	Safe final disposal of infectious wastes includes incineration, open burning in protected area, dump without burning in protected area, or remove offsite with protected storage. If method is incineration, incinerator functioning and fuel available.
	appropriate storage of infectious waste	Observed availability of waste receptacle (pedal bin) with lid and plastic bin liner.
	safe final disposal of sharps	Safe final disposal of sharps includes incineration, open burning in protected area, dump without burning in protected area, or remove offsite with protected storage. If method is incineration, incinerator functioning and fuel available.
	appropriate storage of sharps waste	Observed availability of a sharps container
	guidelines for standard precautions	Observed availability of guidelines for standard precautions anywhere in the facility
	environmental disinfectant	Observed availability of chlorine-based or other country specific environmental disinfection
	latex gloves	Observed availability of latex gloves or equivalent non latex gloves
	disposable of auto-disable syringes	Observed availability of single use syringes (standard disposable or auto-disable)
	soap and running water/ alcohol-based hand rub	Observed availability of soap and running water or alcohol-based hand rub

For each indicator, country-specific estimates of the percentage of health facilities with the service available and across country estimates were calculated with associated 95 % confidence intervals (CI) adjusted for sampling weights. Less than 0.1 % of health facilities were missing data on the indicators considered. To assess the extent of inequalities, estimates were also stratified by facility location (rural, urban) and managing authority (public, private), where data were available. Differences by facility location and managing authority (absolute measures of inequality) were calculated. Chi-square tests were performed to determine if the differences were statistically significant. Due to the variation in categorization of health facility type (e.g. hospital, health center, health post) across countries, surveys and years, differences by type of health facility were not calculated in the present study. Differences between population access and health facility access to water, sanitation and hygiene were also assessed. Country-specific estimates of the percentage of the population with at least basic water, sanitation and hand hygiene services respectively in 2017 were obtained from the WHO/UNICEF Joint Monitoring Program for Water Supply, Sanitation and Hygiene [1]. All analyses and reported estimates adjusted for sampling weights.

## Results

**Fig. 2:**
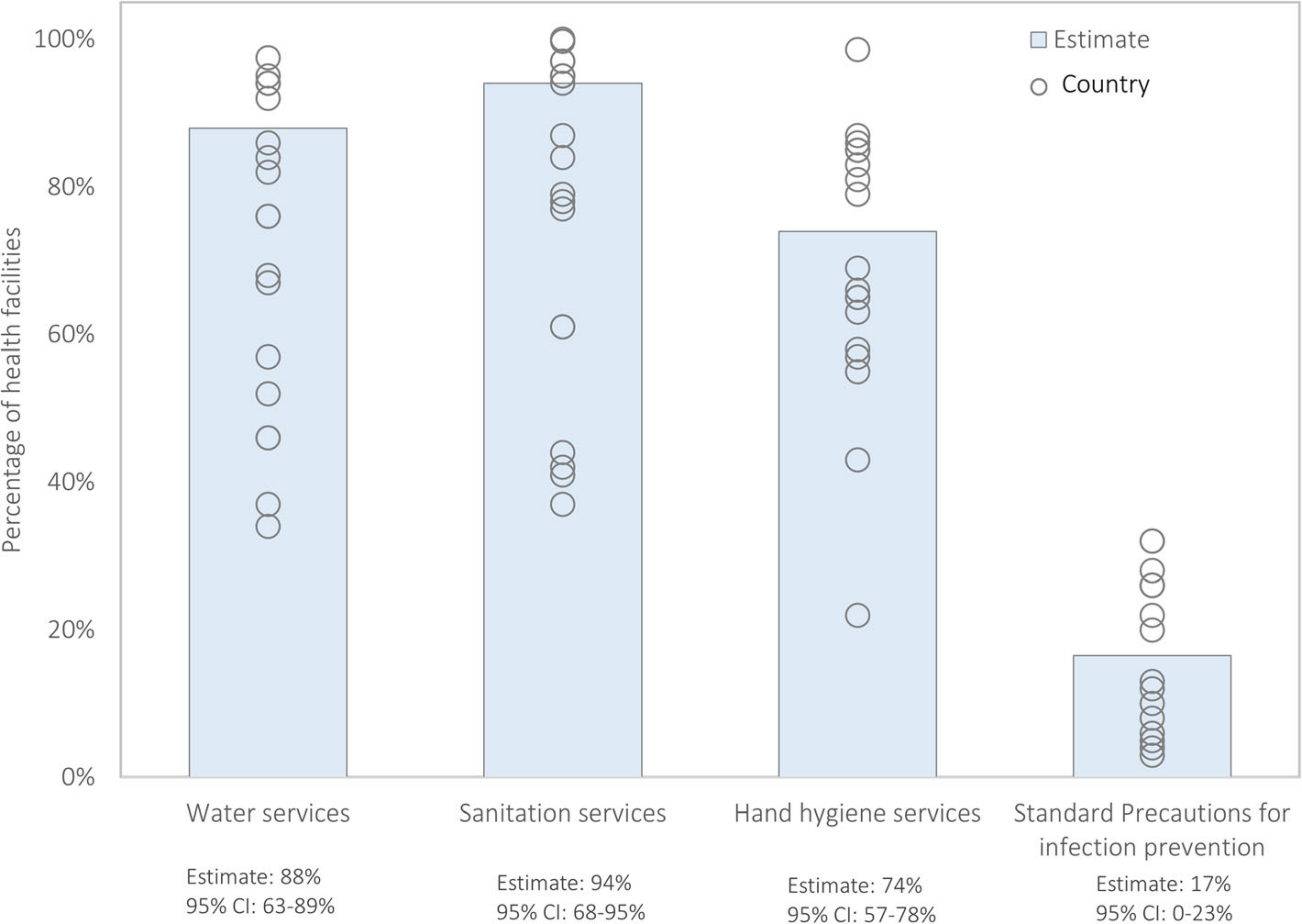
Availability of water, sanitation and hand hygiene services, and standard precautions for infection prevention in health facilities across 18 sub-Saharan African countries

Data was available for 16,456 health facilities from 18 sub-Saharan African countries. The number of health facilities sampled in each country ranged from 206 in Burundi to 2,927 in Kenya (Table 1). Across all countries, 88 % of health facilities had water services defined as an improved water source (95 % confidence interval (CI): 87.1–88.9 %), 94.3 % had sanitation services defined as an improved toilet (95 % CI: 68–95 %), and 67 % had hygiene services defined as soap and running water or alcohol-based hand rub (95 % CI: 65.1–68.7 %; Fig. 2). However, less than a third of health facilities had standard precautions for infection prevention (17 %; 95 % CI: 15.1–19.0 %). Of the tracer items assessed for standard precautions for infection prevention, latex gloves and environmental disinfectant were most commonly available (98.2 and 96.7 % respectively), while guidelines for standard precautions were least available at the time of the survey (46.3 %) (data not shown).

**Fig. 3:**
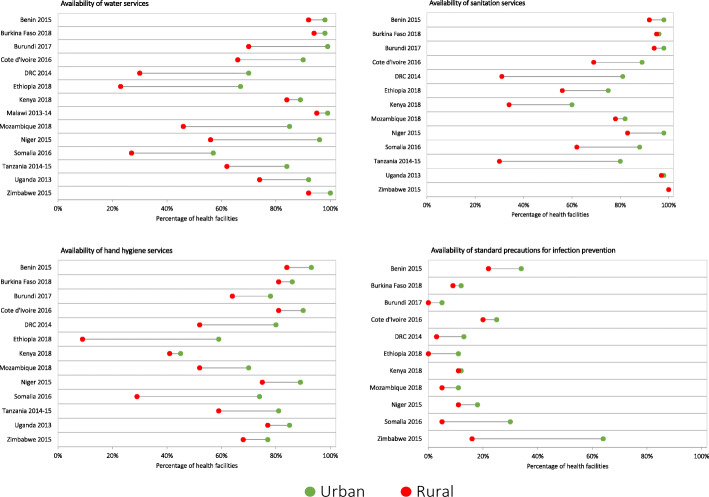
Availability of water, sanitation, and hand hygiene services and standard precautions for infection prevention in rural and urban health facilities

Across the countries with available data on rural-urban classification of health facilities, the availability of water, sanitation and hand hygiene services and standard precautions for infection prevention were generally lower in rural health facilities compared to urban health facilities (Fig. 3). The rural-urban gap in availability of water services in health facilities varied widely across countries, ranging from 4 % points to 44 % points. The difference in availability of water services by rural-urban location was statistically significant (*p* < 0.05). Countries with high availability of water services in health facilities had smaller rural-urban gaps. For instance, Burkina Faso had the highest availability of water services in health facilities across all countries (95 %) and the smallest rural-urban gap (4 % points). By contrast, countries with low availability of water services in health facilities had larger rural-urban gaps. For instance, Ethiopia had the lowest availability of water services at the national level (34 %) and the largest rural-urban gap (44 % points). The magnitude of rural-urban gaps observed in availability of water services were similar to those observed for sanitation services and hand hygiene services. Differences in availability of sanitation services and hand hygiene services by rural-urban location were statistically significant (*p* < 0.05). Standard precautions for infection prevention was least available and had relatively smaller rural-urban gaps that were not statistically significant.

**Fig. 4:**
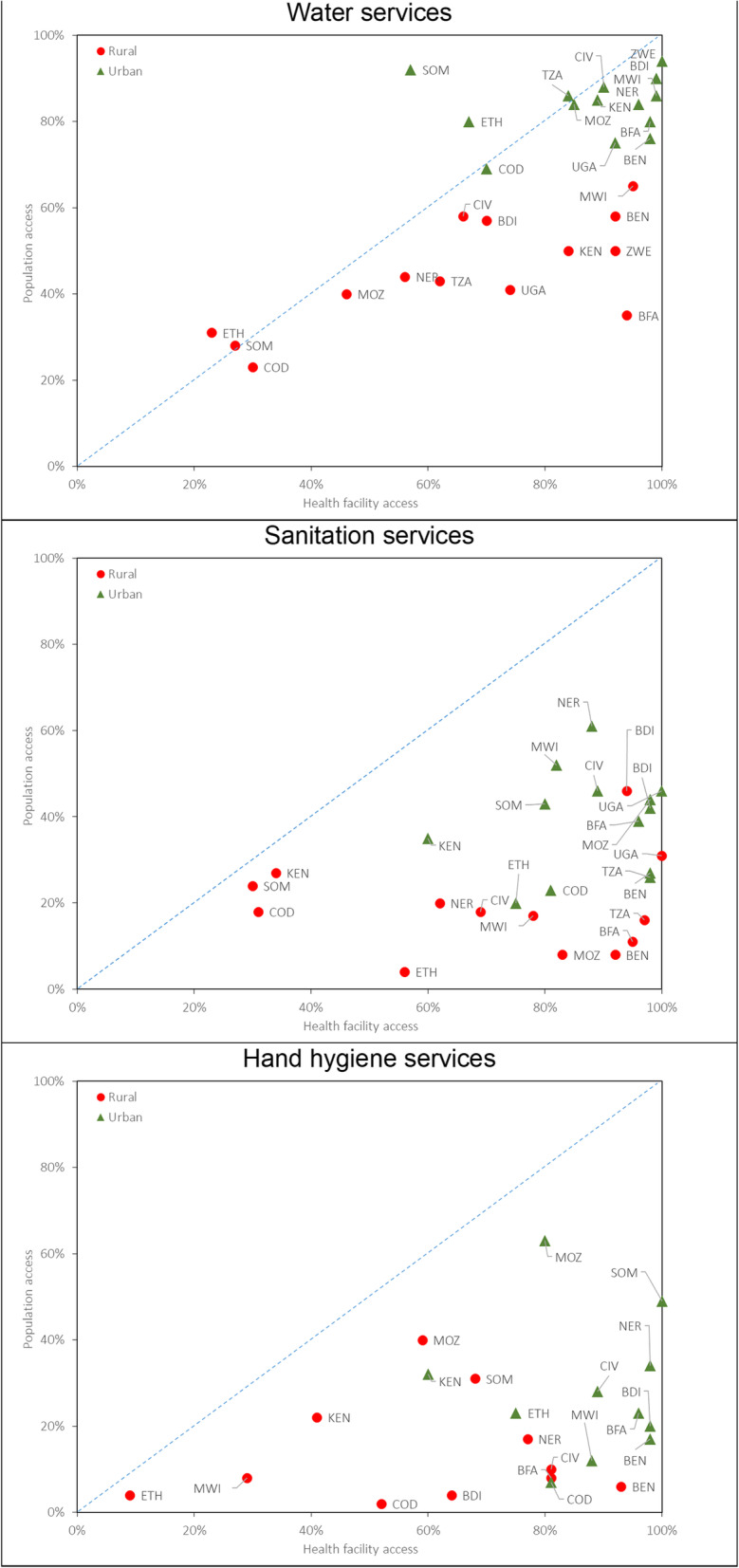
Comparison of population-level and health facility access to water, sanitation, and hand hygiene services

We compared population access and health facility access to WASH services (Fig. 4). Across all countries and all services, both population and health facility access were lower in rural areas than urban areas. With few exceptions (Somalia and Ethiopia), health facility access to water services was consistently higher than population access. Both population and health facility access to water services exceeded 80 % in urban areas in 9 countries. Health facility access to sanitation services exceeded 80 % in urban areas in 12 countries and rural areas in 6 countries, but population access to basic sanitation services was well below 80 % in these same countries regardless of rural-urban setting. Similarly, health facility access to hygiene services exceeded 80 % in urban areas in 9 countries and rural areas in 3 countries, yet less than 50 % of the populations in these areas had access to basic hand hygiene services.

**Fig. 5:**
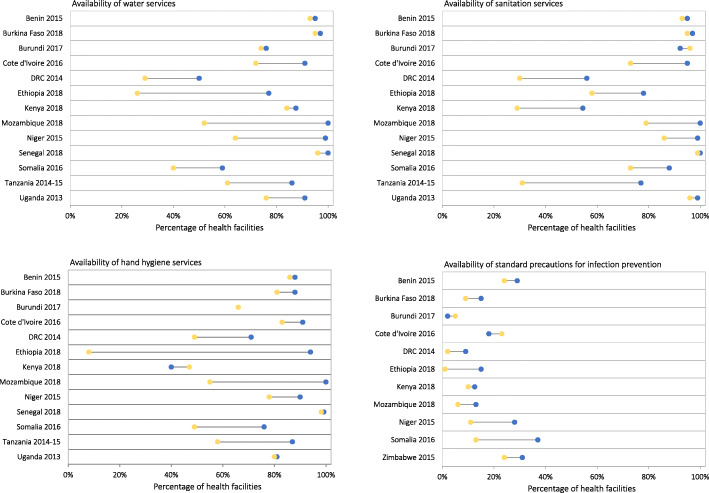
Availability of water, sanitation, and hand hygiene services and standard precautions for infection prevention in public and private health facilities

Compared to private facilities, public facilities were less likely to have access to WASH services (*p* < 0.05) and to a lesser extent, standard precautions for infection prevention available (Fig. 5). However, the advantage of private facilities in access to WASH services was minimal. For Benin, Burundi, and Senegal (< 5 % points). As the availability of standard precautions for infection prevention was low (17 %), differences between public and private health facilities were smaller (Fig. 5).

## Discussion

The overall goal of this study was to assess the availability of WASH services and infection prevention and control measures in health facilities across 18 countries in sub-Saharan Africa between 2013 and 2018. Across countries, an estimated 88 % of health facilities had an improved water source on premises, 94 % had an improved toilet, 74 % had soap and running water or alcohol-based rub available and 17 % had standard precautions for infection prevention available. Our estimates of the availability of water and sanitation services in health facilities were above the global target of 80 % and slightly higher than previously reported regional estimates for 2016, where availability of basic water and sanitation services in health facilities in sub-Saharan Africa was 74 and 71 % respectively [21]. Compared to previously published estimates, the present study included additional countries, used more recent health facility assessments and simultaneously considered health facility and population access to basic WASH services [21, 32]. However, due to insufficient data in the SARA and SPA, this study could not evaluate access to “basic services”. To monitor Sustainable Development Goals (SDG) WASH-related targets in health facilities, WHO/UNICEF Joint Monitoring Program for Water Supply, Sanitation and Hygiene (JMP) has introduced levels of service: “no service”, “limited service” and “basic service” [21]. The criteria used to classify health facilities as offering basic WASH services is more stringent than the definitions used in the present study. There is a need to harmonize core questions and indicators in health facility surveys to contribute to improving access to WASH services in health facilities and accelerate progress towards achieving WASH-related SDG targets.

This study identified gaps in access to WASH services in health facilities by location (rural, urban) and managing authority (public, private). Consistent with previous research, this study demonstrated better access to WASH services in urban health facilities than rural health facilities [21, 32]. This study noted severe deprivation of access to WASH services in rural areas was not only at the health facility level, but at the population level. Rural health facilities constitute a large proportion of the service providers in health systems in sub-Saharan Africa. Furthermore, 59 % of the population of sub-Saharan Africa still resides in rural areas. The disproportionate lack of access to WASH services in rural areas has implications for the prevention and control of infectious diseases and immediate responses to COVID-19.

The current COVID-19 pandemic has disrupted the provision of and reduced access to WASH and waste management services across sub-Saharan Africa. As a result of border closures and lockdowns restricting movement, several countries have reported the disruption of supply chains for hygiene products such as soap and environmental disinfectant as well as equipment such as incinerators [33]. Disruptions in WASH and waste management services in households, health facilities and other public settings have also been documented [34]. For example, water service providers have faced challenges with reductions in revenue, limited availability of resources such as water treatment chemicals and fuel for water pumps, and the lack of maintenance of WASH infrastructure [35]. Households have also faced inconsistent and inadequate access to WASH services, which has the potential to propagate COVID-19 as well as increase the incidence of water-borne diseases. The pandemic has also increased the amount of infectious waste, which, if not appropriately stored and safely disposed, can be detrimental to human health and the environment. Considering the unprecedented impact of the COVID-19 pandemic on the availability of WASH services, several countries have adopted policies and strategies that are locally relevant and based on local resource constraints [36]. In Ghana, the coronavirus alleviation program provided free water including water tankers to vulnerable communities between April and June 2020 [37]. Other countries in the region including Chad, Gabon, Guinea, Kenya, Mauritania, and Togo have implemented similar social protection plans to offer utility waivers for water and electricity for all or part of the population amid the COVID-19 pandemic [38, 39]. However, these provisions miss unconnected households that are the most vulnerable [39]. Several countries have focused on longer term solutions such as the construction of sanitation facilities and handwashing stations in households, health facilities, transportation hubs and markets, the provision of WASH and electricity services in high priority settings and the local production of and solidifying supply chains for WASH commodities such as soap and hand sanitizers [40, 41]. Whether these innovative solutions will be implemented at scale and remain accessible to populations in an equitable manner is yet to be determined.

There are several limitations worth noting. Estimates of the availability of WASH and standard precautions for infection prevention in health facilities across sub-Saharan Africa were based on health facility surveys conducted between 2013 and 2018. First, the estimates likely do not reflect the current situation in health facilities, given intermittent or discontinued supplies and stock-outs that are widespread in the region [42, 43]. Further, the current pandemic is likely to impact supply chains and the implementation of infection prevention control measures. However, assessing the situation prior to the pandemic has the potential to inform strategies and interventions needed to interrupt COVID-19 transmission. Second, because of lack of recent health facility survey data, the study does not include all sub-Saharan African countries. For a comprehensive understanding of the status of health facilities, more frequent health facility assessments such as the SPA and SARA are needed. Also, the harmonization of data collection tools and indicators will allow the monitoring and evaluation of progress at national, regional and global levels. Third, the study does not capture all aspects of WASH services in health facilities (e.g. acceptability, quality, quantity and accessibility). The study also does not capture adherence to WASH, waste management and infection prevention protocols in the health facilities. Poor infection prevention and control practices may render health facilities as vehicles of transmission, similar to what has previously been observed with Ebola [44]. A recent health facility assessment in Tanzania found that adherence to infection prevention and control measures, particularly hand hygiene was inadequate [45]. Given frequent handwashing with soap and water is one of the most important COVID-19 prevention measures, adherence to safety precautions and engagement in appropriate WASH-related behaviors has likely changed in response to the pandemic. Fourth, while the study assessed inequalities by location and managing authority, other relevant factors such as access to health facilities by different populations were not accounted for. Moreover, while the study provides an initial understanding of differentials in access by location and managing authority, health facilities located in the same setting or managed by the same authority are not uniform. Assessment of rural-urban inequalities may mask the situation in health facilities in urban slums, where populations also have poor access to WASH services. Lastly, estimates of population access to WASH services were obtained for 2017 which in some cases differs from the year the health facility survey (SPA or SARA) was conducted.

Despite these limitations, this study leverages the SPA and SARA data allowing a standard approach for comparisons within and between countries. The data used represents 18 sub-Saharan African countries with varying population characteristics and social determinants of health. Improving access to WASH, health care waste management and environmental cleaning is essential for the delivery of quality health services, and the prevention and control of infectious diseases. Containing the pandemic will depend on a multi-pronged approach that targets all settings such as households, health facilities, schools and workplaces. Amid the COVID-19 pandemic, ensuring adequate access to WASH services and enforcing adherence to infection prevention and control practices both at health facility and community levels by governments and relevant institutions remains crucial to minimizing transmission risks.

## Data Availability

All SPA data are available to download free of charge by registered users from the DHS Program at https://dhsprogram.com/data/. Dataset access for the SARA can be requested at http://apps.who.int/healthinfo/systems/datacatalog/index.php/catalog.

## Abbreviations

COVID-19: Coronavirus disease
LMIC: Low- and middle-income countries
PPE: Personal protective equipment
SARA: Service Availability and Readiness Assessment
SDG: Sustainable Development Goal
SPA: Service Provision Assessment
WASH: Water, sanitation and hygiene
WHO: World Health Organization
